# Initial coin offerings (ICOs): Why do they succeed?

**DOI:** 10.1186/s40854-021-00317-2

**Published:** 2022-01-17

**Authors:** José Campino, Ana Brochado, Álvaro Rosa

**Affiliations:** 1 Instituto Universitário de Lisboa (ISCTE-IUL), Business Research Unit (BRU-IUL), Lisbon, Portugal; 2 Instituto Universitário de Lisboa (ISCTE-IUL), DINÂMIA’CET, Lisbon, Portugal

**Keywords:** Initial coin offering (ICO), Fintech, Bank, Financial services, Technology, Blockchain, Innovation, Venture capital, Crowdfunding, Success

## Abstract

Recent literature has addressed initial coin offering (ICO) projects, which are an innovative form of venture financing through cryptocurrencies using blockchain technology. Many features of ICOs remain unexplored, leaving much room for additional research, including the success factors of ICO projects. We investigate the success of ICO projects, with our main purpose being to identify factors that influence a project’s outcome. Following a literature review, from which several potential variables were collected, we used a database comprising 428 ICO projects in the banking/financial sector to regress several econometric models. We confirmed the impacts of several variables and obtained particularly valuable results concerning project and campaign variables. We confirmed the importance of a well-structured and informative whitepaper. The proximity to certain markets with high availability of financial and human capital is also an important determinant of the success of an ICO. We also confirm the strong dependency on cryptocurrency and the impact of cryptocurrency valuations on the success of a project. Furthermore, we confirm the importance of social media in ICO projects, as well as the importance of human capital characteristics. Our research contributes to the ICO literature by capturing most of the success factors previously identified and testing their impacts based on a large database. The current research contributes to the building of systems theory and signaling theory by adapting their frameworks to the ICO environment. Our results are also important for regulators, as ICOs are mainly unregulated and have vast future potential, and for investors, who can benefit from our analysis and use it in their due diligence.

## Introduction

Initial coin offerings (ICOs) are a novel concept that first appeared in 2013 with the MasterCoin project proposed by J. R. Willett. These projects allow the financing of innovative ideas at a global level, which contributes to the democratization of financial investments and allows an entire new reach hardly achieved through conventional means (Brochado 2018). Highly technological solutions and the role of e-business have gained paramount importance, especially as a strong contributor to keeping the economy running in an extreme scenario, such as during the Covid-19 pandemic (Al-Omoush et al. 2020). Initial coin offering projects are technological ventures based on blockchain technology and are financed using cryptocurrency (Massey et al. 2017). An investor must convert fiat currency into cryptocurrency to participate in the project (Kranz et al. 2019). Once the funds have been released to the project promoter, the investor must receive the tokens corresponding to the contribution made. Tokens can assume different forms in ICOs (Howell et al. 2018): (i) currency tokens, such as cryptocurrency, for exchange and to be stored; (ii) security tokens, used as a traditional security but backed by a blockchain infrastructure; (iii) utility tokens (the most used type of token), which grant the investor rights to access a product or service.

The characteristics of the project are compiled in a whitepaper, which is unregulated but tends to follow certain characteristics and can be compared to a regulated prospectus. The whitepaper is also a measure of the project’s credibility, as it contains technical information, business information, and information regarding the team.

As in crowdfunding, the success of an ICO project may depend on the amount of capital raised. An ICO project may have none to several thresholds defining the capital to be raised (Kranz et al. 2019): (i) no-cap: project without any limits regarding financing; (ii) soft-cap: minimum limit of capital achieved, in order to proceed with the project; (iii) hard-cap: maximum amount of capital accepted; (iv) collect and return: a hard cap is defined and, if surpassed, the tokens are distributed with respect to the ratio of the hard cap to the total funds received; (v) dynamic ceiling: several hard-cap limits are defined and kept secret; (vi) a combination of several of these characteristics. Initial coin offerings can be defined as a “decentralized method of financing, whereby a firm calls for funding by issuing coins to online investors. Coins (or tokens) are a digital medium of value exchange based on the blockchain, which can operate independently and can be traded between investors” (Huang et al. 2020, p. 3). This definition includes one essential characteristic of ICO projects, namely, the existence of a secondary market for the tokens sold.

Interest in digital solutions has increased, particularly during times of isolation, such as the Covid-19 pandemic. This can be seen in the strong correlation between the price of gold in an economic downturn and the valorization of ESPO, an exchange traded fund that tracks the market for the gaming industry (López-Cabarcos et al. 2020). Similarly, ICOs can benefit from their digital characteristics and gain the interest of the public. Indeed, interest in ICO projects has increased, as revealed by internet search trends and the size of the ICO market following that trend (Google 2020). From 2016 to 2019, 1676 token sales were successfully concluded, amounting to a total of approximately USD 29.2 billion (Coinschedule 2020). The highest-financed project obtained USD 4.1 billion—a 2017 project named EOS, which is software based on blockchain technology (ICOBench 2020). The countries with the highest number of ICOs and the greatest amount of capital are the USA and Singapore. In terms of the number of ICOs, the UK is in third place, and in terms of capital raised, the British Virgin Islands are in third place (ICOBench 2020). The categories in which ICOs are employed vary from year to year, but investment in blockchain infrastructure is a constant investment regardless of the year. In 2019, the majority of ICO investments were in trading and investment platforms, payment platforms, and blockchain infrastructure (Coinschedule 2020). The hype gained by ICO projects fell in 2019 following two years of considerable investment and high numbers of token sales successfully concluded. This fall is also due to the depreciation of cryptocurrency values in 2019 after two years of enormous appreciation against fiat currencies (Fisch 2019).

Initial coin offering projects have been compared to other more traditional forms of financing, such as initial public offerings (Ofir and Sadeh 2019), venture capital (VC), and crowdfunding (Block et al. 2020). Nevertheless, ICOs have unique characteristics that differentiate them from other types of financing (Biasi and Chakravorti 2019) and make them more suitable for financing innovative projects that offer information goods (Chod and Lyandres 2020). Indeed, the use of blockchain solutions (e.g., equity-based security token offerings) for financing new ventures has several advantages. Regardless of the sector in which they operate, newly created ventures obtained significantly lower discount rates when using these solutions compared to VC financing (Pazos 2019), increasing entrepreneurs’ returns (Catalini and Gans 2018). These unique characteristics are, among others, much lower costs involved in the investment process, investment in cryptocurrencies, projects based on blockchain technology, existence of a secondary market for the tokens sold (Chen 2018), no third parties involved, and lower investment thresholds (OECD 2019). These characteristics allow the democratization of access to capital markets (Brochado 2018).

Democratization shows the global reach of these projects. Indeed, ICO projects use the power of a wide reach to obtain contributions from a vast crowd of investors. As in crowdsourcing, ICO projects can reduce or eliminate the involvement of third parties because of the blockchain’s trust-free (participants no longer need a trusted third party) and transparent nature, which allows completely secure transactions (Frizzo-Barker et al. 2019). The use of the crowd and its direct link to entrepreneurs or companies can provide several benefits, namely, reduction of costs, greater brand visibility, and access to specialized skills (Christensen and Karlsson 2019).

The current research focuses on the success factors of ICO projects, since the literature to date on this is still scarce and has several gaps (Chen and Chen 2020). To the best of our knowledge, there are no studies capturing an extensive range of success factors; most focus on particular impacts of specific variables. Our study identified several success factors and grouped them into categories. Thus, we contribute to the literature by grouping a large number of success factors into a single investigation applied to a single large database. We focused on the characteristics of projects that influence their final outcomes and determined which of them are the most important. Our research aimed to answer the following proposition: What are the success factors of ICO projects and what are their impacts on project outcomes?

## Literature review

### Theoretical background

#### The general systems theory

The general systems theory has its roots in the 1940s, when the Austrian biologist Ludwig von Bertalanffy created a new approach applicable to all fields of science that sought to address the increasing complexity of the world’s problems. The main objective of this theory is to understand the individual and his environment as part of a broad interactive system, and the aim is to study the interactions from different holistic perspectives (Skyttner 2005). In short, the theory sees each activity, object, or individual as not behaving alone, but as being part of a larger system with which they interact.

A system can be understood as a combination of objects that have regular interactions and are interdependent (Mele et al. 2010). The theory seeks to deconstruct every objectof study to understand the system in which it is integrated. It is argued that it is impossible to understand a given phenomenon only by analyzing its elementary components, but rather by observing it from a higher level (Mele et al. 2010).

The role of information is also highlighted in systems theory, as it is particularly important in communication in accordance with information theory. The flow of information should be from the sender to the receiver, who then provides crucial feedback on the continuity of the flow (Bertalanffy 1968). An important principle related to information is the distinction between open, closed, and isolated systems. In open systems, the participants exchange energy, matter, people, and information, whereas in closed systems, there are exchanges of only energy, and in isolated systems, there are no exchanges (Mele et al. 2010). Open systems theory (OST) builds on these concepts to look at organizations and their relationships with the environment in which they operate. It is argued that organizations must be able to process information about their environment and adapt, in order to thrive. Therefore, organizations must be adaptable, in order to survive (Katz and Kahn 1979).

The study of cybernetics highlights that the actions taken by a system can cause changes in the environment. This leads to the adaptation of the system, as changes in the environment are perceived as feedback (Skyttner 2005; Mele et al. 2010). The last important concept to address in this study concerns the idea suggested by the viable system approach. This approach states that there are relationships among sub-systems (internal components of the organizations) and supra-systems (organizations themselves and other systemic entities) (Mele et al. 2010).

Initial coin offering projects can be classified as ventures operating in an open systems model, similar to crowdsourcing (Geiger et al. 2011a). As in crowd funding and crowdsourcing, ICO ventures have several iterations with external agents, which can alter the project’s perception (Zha et al. 2020), namely, the website where the ICO is published, specialists who classify the project by providing ratings, and several stakeholders who are ultimately potential investors (this iteration can occur before, during, and after the financing campaign). Blockchain projects can facilitate governance functions (e.g., voting and coalitions), as entrepreneurs and investors can be involved in corporate governance matters to protect their interests. This proves the true nature of ICOs as an open system (Catalini and Gans 2018; Frizzo-Barker et al. 2019).

This interaction with external parties is characterized by an exchange of information that generates stimuli used in business processes to improve or reward investors (Doan et al. 2011). Similarly, the crowdsourcing projects and ICO projects have a process that proves their operation within an open system and as part of a larger environment: (i) the preselection of contributors, (ii) accessibility of peer contributions, (iii) aggregation of contributions, and (iv) remuneration for contributions (Geiger et al. 2011b). The relationships between the systems in ICOs are long lasting, as they continue after the project is completed and the token advances to the secondary market (Ackermann et al. 2020). The iterations should provide feedback that allows the system to adapt (Bertalanffy 1968). Hence, feedback is of tremendous importance in ICOs because of the role of the investors’ network and their presence in social networks (Zha et al. 2020), where good feedback can lead to further investment (Xuan et al. 2020).

According to systems theory, organizations themselves are seen as systems that are composed of subsystems divided into two components: (i) social components, which represent the people, and (ii) technical components, which represent the technology and machines (Emery and Trist 1965). There are several iterations among subsystems (Mele et al. 2010). Initial coin offering projects are characterized by their core technological component (Albrecht et al. 2019), but the importance of the team and its characteristics has become important in the literature (An et al. 2019). The relationship between the social and technological components should be analyzed as part of a successful project. Indeed, the technological aspects of a project may be complemented by aspects of the team and its characteristics.

The supra-system relationship in ICOs can be challenging because of the lack of regulation and surveillance, which can lead to a lack of transparency concerning the project’s details (Hacker and Thomale 2018; Giudici and Adhami 2019). Indeed, blockchain projects allow privacy and transparency, as the information on a certain transaction is made public but the parties involved are kept private. Some challenges and risks associated with blockchain projects are the volatility of cryptocurrencies, governance, regulation, fraud, environmental costs, and security, as there is a guarantee that the information coded is true (Frizzo-Barker et al. 2019). Therefore, information assumes a crucial role, as described by systems theory. Initial coin offerings should be able to process and send information, as described by OST, and then receive and incorporate feedback. Organizations that are able to survive in a certain context characterized by change by adapting to the feedback received are considered viable systems (Mele et al. 2010). The exchange of information described here can be materialized in the signals sent by the ICO projects to investors in this supra-system relationship (connection among enterprises). Investors should then send feedback to projects that inform them of good or poor performance. This should lead to the adaptation of projects in this constant open systems relationship. As the signals described here assume a crucial role in the supra-system relationship, signaling theory might contribute to the analysis undertaken in the current research.

#### Signaling theory

Initial coin offerings must be understood as part of an environment comprising several systems. These systems interact with each other and within themselves. The supra-system interactions are characterized by the exchange of information, here treated as signals of the projects’ quality and posterior feedback. However, the ICO market is characterized by information asymmetries between promoters and investors (Momtaz 2019). The promoters of the projects hold crucial information on their own capabilities and each project’s characteristics, which investors do not (Yadav 2017). Building on signaling theory, this is an information asymmetry problem (Spence 1973). Signaling theory states that several markets are characterized by an information gap between buyers and sellers, notably financial markets, in which investors do not have the same level of information as entrepreneurs.

Building on signaling theory, another issue is raised concerning the high information disparities between the project’s promoters and investors during supra-system interactions. The issue concerns the adverse selection concept, which states that when there are considerable differences in the level of information between two agents interacting with one another in an open systems relationship, the one with more information can proceed in a dishonest way, to the disadvantage of the other. An agent with less information is not able to understand the true quality of a product (Wilson 1989). In a market characterized by adverse selection, there is no distinction between good-quality projects and poor-quality projects that are sold at the same price (Grossman 1981). Therefore, there is a need to ensure that buyers with less information are provided with quality signals that can identify a good quality product in a very unequal market (Milgrom 1981).

Without proper information transfer among the participants, the markets will perform poorly, as entrepreneurs may not always be completely transparent in the information they provide. Performing correct and deep due diligence is costly; thus, third parties arise, in order to overcome this difficulty. Being an outside party connecting both investors and entrepreneurs, these institutions fulfill the role of collecting unbiased information and are the (remunerated) channels among market participants (Leland and Pyle 1977).

Thus, signaling theory relies on signalers, receivers, and signals. Signalers have access to privileged information and must transmit it to the receivers, in order to be perceived as having a high-quality project. The signals sent to the receivers must have two characteristics, in order to be effective in reducing information asymmetry (in the event that it exists): (i) be observable to the receiver; (ii) be costly to realize and imitate, since if no costs are involved, the signals will be easy to replicate and, thus, have no value (Domingo et al. 2020). A crucial assumption in signalling theory is that equivalent signals have different costs depending on high- or low-quality projects. If the costs to produce a signal are much higher in a low-quality project than in a higher quality project, only the latter will choose to produce them.

In VC projects, it is assumed that the aspects to be confirmed in the due diligence process are: (i) the size of the problem that the business is attempting to solve, (ii) the elegance of the solution, (iii) the entrepreneurial team, (iv) financial statements, and (v) legal aspects (Yadav 2017). As ICO projects are highly technological, due to using distributed ledger technology (Kranz et al. 2019), they come with enormous financial risk (Kou et al. 2014); they include a lack of information disclosure and information asymmetries are substantially greater for these projects, which increases the need for signalling (Fisch 2019).

As ICOs are highly technological, signals they might use to affirm high-quality projects to investors are: (i) patents (which are crucial in early financing stages and fulfill the criteria for effective signalling); (ii) technical whitepaper (as the primary source of detailed information about the project, the whitepaper should have information on the technological infrastructure of the project that is costly to produce and explain); (iii) high-quality source code (most developments based on blockchain technology occur through programming and, thus, high-quality code is required) (Fisch 2019). Signals to reduce information asymmetries might be published in the whitepaper, but they might also be available in other sources, such as dedicated ICO websites with extensive databases (Giudici and Adhami 2019) or social networks, such as Twitter (Xuan et al. 2020) or GitHub (Roosenboom et al. 2020).

### Success factors

Signals are considered to be success factors of ICO projects because they reduce information asymmetries and the projects are more easily perceived as high-quality projects (Ackermann et al. 2020). There are additional factors that may not be considered signals because they do not fulfill the necessary characteristics, but they can also influence the success of a project. Attention in the past was mainly directed toward crowdfunding projects, simply because they predated ICO projects. The literature reports many attempts to adapt crowdfunding success factors to ICO projects because of their similarities. Nevertheless, the success factors that have been identified as relevant in both traditional and blockchain-based crowdfunding are few and are built around the following features: (i) industry, (ii) location, (iii) team size, (iv) number of advisors, (v) social network presence, (vi) share of retained equity/tokens, and (vii) early investment possibility (Hartmann, Grottolo, Wang, & Lunesu, 2019). Success factors can be categorized, for instance, as being related to the project itself or the campaign, and the literature has paid special attention to the importance of social networks as a determinant success factor in these types of project (Albrecht et al. 2019). Team characteristics have also received attention (Giudici and Adhami 2019).

#### Success factors of the project

The project’s success factors cover characteristics inherent to the project itself, namely, every characteristic is predefined when the ICO starts and is related to the idea proposed and the future outcome. In crowdfunding, technological companies typically obtain the most financing and are also the most successful. Of these, younger companies are the most successful because crowdfunding generally targets these companies specifically (Ralcheva and Roosenboom 2016). As they are also a way of financing high-risk projects, ICO projects fulfill these characteristics because they are technological ventures based on blockchain, mostly without any track record, and are created only to conclude the ICO and develop a project.

The first success factor we identified was related to the industry in which the project was positioned. The project’s industry is directly linked to its success, and studies reveal that, depending on the project’s area, there are different coefficients influencing the outcome of the project, some of which negatively influence the outcome (Davies and Giovannetti 2018). Note that the project promoters’ previous experience in the industry is not considered relevant for a successful outcome, revealing that entrepreneurial experience and industry expertise are not necessary for conducting a successful project (Mamonov and Malaga 2018).

The location factor is also widely considered in the literature and has been reported to project success (Davies and Giovannetti 2018; Charlotte et al. 2019; Fisch 2019; Ackermann et al. 2020). The geography of ICOs has been found to be important because its presence in markets with specific conditions contributes to the flourishing of blockchain-based ventures. For instance, the existence of developed financial markets, advanced digital technologies, crowdfunding platforms, and the will to develop regulations contribute significantly to the attraction of ICO projects (Huang et al. 2020). Projects located in the USA are considered to have particularly positive outcomes, (Fisch 2019), along with projects located in Israel and China (Fenu et al. 2018). It is also suggested that projects located in larger cities are more successful than others (Ralcheva and Roosenboom 2016).

In ICO projects, a reference to follow specific legislation does not influence the project’s success, mainly because of the paucity of regulation in the market (Giudici and Adhami 2019). As the overall ICO market is unregulated, investors tend to reduce information asymmetries through the channels available to them, namely, the project’s website, social media platforms, and whitepapers. As a result, opaque projects are disfavored by investors and are less successful (Bourveau et al. 2018). Despite the lack of regulation, investors follow certain rules that could help regulators intervene in the future (Amsden and Schweizer 2019). An increase in the regulation and supervision of the ICO market is expected, due to the importance of investor protection and crime/fraud prevention. These measures may contribute to the expansion of the ICO market (Huang et al. 2020).

Two concrete proposals for ICO market regulation are as follows: (i) international efforts for the creation of international legislation concerning ICOs; (ii) EU regulatory actions to make ICOs comply with regulatory guidelines concerning the disclosure of crucial information (Hacker and Thomale 2018). Although not regulated and not following specific guidelines, most of a project’s formal information is described in whitepapers, which are a crucial step in an ICO project. Several studies have focused on the role of the whitepaper as a fundamental way of reducing information asymmetry and have arrived at some important conclusions. Nevertheless, the mere existence of a whitepaper does not positively influence a project’s outcome; importance resides in the content of the whitepaper (Adhami, Giudici, & Martinazzi, 2018). There is a common idea that the length of the whitepaper influences a project’s success (Bourveau et al. 2018; Amsden and Schweizer 2019; Fisch 2019). Being a primary source of information, its greatest value resides in reducing information asymmetry between promoters and investors (Ofir and Sadeh 2019).

Although the conclusions are very similar concerning the length of the whitepaper, there are some discrepancies concerning the technical nature of the whitepaper. Technical whitepapers include those in the areas of system architecture, smart contract description, and technical diagrams (Albrecht et al. 2019). Whitepapers with technical aspects are considered to contribute to the success of the project because they are generally taken as a sign of quality and technical expertise (Feng et al. 2019; Fisch 2019). Nevertheless, there are arguments on the opposite side, assuming that technical whitepapers do not influence a positive outcome in the long term, but have a positive impact only at the beginning of the ICO campaign (Albrecht et al. 2019).

Having a secondary market and a tradable token is a predominant characteristic of ICO projects and the main distinguishing point from crowdfunding (Brochado 2018). Having a secondary market, that is, being listed on at least one crypto exchange, is crucial to a project’s success. As the tradability of the token in the secondary market is of tremendous importance (Ackermann et al. 2020), there is also a positive effect from being listed in more than one crypto exchange (Lyandres et al. 2019). Some researchers consider the secondary market to be as important as the capital raised and it is itself, therefore, considered to be a measure of success because the project is appraised as successful only once it is tradable (Amsden and Schweizer 2019). It is important to highlight that token value is highly volatile and can be seriously jeopardized by adverse industry events, such as technical hacks or regulatory actions (Momtaz 2020a).

#### Success factors of the campaign

The campaign’s success factors are focused on the relevant features prepared prior to the beginning of the campaign and during that period. These factors are of enormous importance, as the campaign period is the timeframe during which promoters raise capital and is when the project reveals a positive or negative outcome (achieving or not achieving the minimum threshold of capital needed). The common argument is that a longer campaign negatively affects the performance of a project, and shorter campaigns are more likely to see better outcomes (Davies and Giovannetti 2018; An et al. 2019; Fisch 2019; Ackermann et al. 2020; Roosenboom et al. 2020). It is also important that, during the campaign period, promoters do not put excessive pressure on investors to obtain financing because these attitudes are associated with negative results in terms of capital raised (Albrecht et al. 2019).

Before the campaign’s official period starts, it is very common to have a pre-sale of tokens in ICO projects. These sales offer discounts and bonuses to investors who bear more risk by making an early investment (Liu and Wang 2019). Several studies suggest a positive impact of pre-sale campaigns and project success (Giudici and Adhami 2019; Lyandres et al. 2019; Ackermann et al. 2020; Roosenboom et al. 2020). There is also a concern that pre-sale campaigns may indeed have a negative impact on a project’s success (Momtaz 2020a), mainly because investors perceive an immediate need to cover expenses, and the bonuses offered to investors may lead to them dumping the tokens in the secondary market, in order to maximize profits, thereby negatively impacting the project’s overall success (Amsden and Schweizer 2019). The bonus schemes, sometimes offered by the promoters, may negatively affect a project’s success (Adhami et al. 2018; Charlotte et al. 2019; Giudici and Adhami 2019; Roosenboom et al. 2020) because ICO projects with larger bonuses are perceived as possible scams (Lee et al. 2019), increasing the chances of token dumping in the secondary market. Meanwhile, lower token prices seem to have a positive influence on the project’s success because investors tend to be more interested in cheaper tokens, allowing them to buy several tokens from different projects (Burns and Moro 2018; de Jong et al. 2018; Yuryev 2018). The need to buy tokens from different projects concerns the need to have portfolio diversification because, due to information asymmetries, investors have a high likelihood of selecting poor projects, which underscores the importance of portfolio diversification (Boreiko and Risteski 2020).

Nevertheless, most of the time investors can spot scam projects and avoid investing in them, which provides them with tremendous returns when investing in an ICO (Benedetti and Kostovetsky 2018). In terms of a project’s financing thresholds (i.e., soft-cap and hard-cap limits), the existence of hard-cap limits positively influences the project’s success, as investors can better assess the value of the tokens (Amsden and Schweizer 2019). However, higher hard-cap limits, seemingly impossible to achieve, have negative effects on the project’s success (Lyandres et al. 2019). The existence of soft-cap limits is considered to have a positive influence on a project’s success (Amsden and Schweizer 2019), but there is no consensus, as there is also evidence of its negative effects (Bourveau et al. 2018).

Investment in ICOs is done using cryptocurrency, and several cryptocurrencies may be accepted by the promoters of a project. Campaigns accepting multiple currencies are more successful than those that accept only one (Charlotte et al. 2019; Lee et al. 2019). Accepting several currencies is evidence of a project’s quality, revealing technical knowledge, since accepting numerous currencies requires blockchain expertise (Amsden and Schweizer 2019). There are concerns regarding the huge volatility of cryptocurrencies (Frizzo-Barker et al. 2019).

As ICO financing is done via cryptocurrencies, their volatility has a large impact on a project’s success, particularly Ethereum volatility (Myalo and Glukhov 2019), because most projects are based on Ethereum technology (Fenu et al. 2018). Not surprisingly, Ethereum-based ventures achieved more successful results (Fisch 2019). Consequently, the higher price of Ether diminishes the attractiveness of investment in an ICO, which means a higher opportunity cost for the investor, and it is, thus, negatively correlated with the project’s success (Amsden and Schweizer 2019; Roosenboom et al. 2020).

Most projects require quality code to be successful and to smoothly meet the many requirements of an ICO campaign. Therefore, the existence and availability of high-quality code or code parts positively influence a project’s success (Blaseg 2018; Amsden and Schweizer 2019; Ackermann et al. 2020) because investors have the chance to assess the degree of the project’s technical quality (Adhami et al. 2018).

Finally, experts’ ratings of projects are also a way of reducing information asymmetries and identifying better projects. Although some concerns have been raised, the ratings attributed by third parties tend to replace traditional third-party involvement and can determine the success of an ICO with considerable precision (Liu and Wang 2019). Consequently, experts’ ratings are linked to project success (Fenu et al. 2018; Rhue 2018; Lee et al. 2019; Xuan et al. 2020).

#### Success factors of social networks

Social networks are strong contributors to innovation through the promotion of collaborative work (Al-Omoush et al. 2020). Great weight has been given to the presence and use of social networks, as they influence the output of ICO projects. Social networks are key to conferring legitimacy to projects, as the content provided in the social networks’ environment is not entirely controlled by project promoters. Indeed, media-provided content is more effective than firm-provided content in influencing investors’ behavior (Chanson et al. 2018). Skillful management of social networks can promote high amounts of early contributions and the constant updates throughout the ICO campaign contribute to a project’s success (Ante et al. 2018; Bourveau et al. 2018; Ackermann et al. 2020).

Social media can be used to influence investors’ behavior (Liu and Wang 2019), but simply being present on one or several social networks does not influence the project’s success. In order to be relevant, social networks must be maintained and managed correctly, including the frequent posting of updates on the campaign (Xuan et al. 2020). The importance of social media derives from the fact that it can influence investors’ behavior (Liu and Wang 2019). The most commonly used social networks in ICO projects are Twitter and GitHub, with the latter being a public repository of code. Concerning Twitter, there is evidence of a positive relationship between market capitalization and the active use of Twitter. Nevertheless, this utilization must be regular and not exaggerated, since high-intensity activity on Twitter is associated with positive returns in the very short term, but with negative returns beyond that (Benedetti and Kostovetsky 2018). Activity on Twitter must be related to positive messages associated with the project, and interactions with potential investors must be maintained during the campaign (Albrecht et al. 2019). The content related to the Twitter account is of paramount importance, but some content may be interpreted as nothing more than cheap marketing and have negative effects on the project’s success—for instance, linking the campaign with the cryptocurrency topic or to the blockchain topic (Albrecht et al. 2019).

There is proof that the activity of the social network account is also important because there is a proven negative effect on the number of accounts the project is following and the project’s success (Albrecht et al. 2019). This is also considered to be cheap marketing and an easy way to obtain followers. Overall, the importance of having a Twitter account in ICO projects is clearly demonstrated, as this is a good way to better communicate with investors and an additional way to reduce information asymmetries (Burns and Moro 2018; Cerchiello et al. 2019; Fisch 2019; Xuan et al. 2020), similar to what happens in crowdfunding (Greenberg et al. 2013).

The importance of having active and well-managed social network accounts is extended to the use of the GitHub platform as a source of public code, which strengthens the project’s success (Albrecht et al. 2019; Amsden and Schweizer 2019), especially during the pre-sales of tokens (Roosenboom et al. 2020). Finally, several researchers point to the importance of having an active and well-managed website as a driver to differentiate high-quality projects from scams, which also contributes to positive project outcomes (de Jong et al. 2018; Cerchiello et al. 2019; Pereza et al. 2020).

#### Success factors of the team

Social capital has been identified as pivotal in the development of companies’ proactiveness. Furthermore, social capital is crucial for the development of entrepreneurial and innovative digital opportunities (Al-Omoush et al. 2020). The importance of a team’s characteristics and its disclosure has been highlighted in the literature as having an impact on a project’s success (An et al. 2019). Several human capital characteristics have been highlighted, including team size, professional experience, technological background, and social media presence (Brochado 2018). In ICO projects, larger teams tend to be related to more successful projects, as pointed out in several studies (Ante et al. 2018; Amsden and Schweizer 2019; Cerchiello et al. 2019; Giudici and Adhami 2019; Liu and Wang 2019; Roosenboom et al. 2020). Similarly, there is a positive relationship between larger advisory teams and the success of ICO projects (Ante et al. 2018; Amsden and Schweizer 2019; Cerchiello et al. 2019; Charlotte et al. 2019; Giudici and Adhami 2019). Concerning team characteristics, such as professional experience, managerial experience, education, and entrepreneurial background, there is proof that only past managerial experience is relevant for a project’s success, while education, professional experience, and entrepreneurial background are not relevant (Giudici and Adhami 2019). The same has been demonstrated in crowdfunding projects (Allison et al. 2017). Likewise, in human capital characteristics, higher ratings attributed to teams by independent experts are associated with successful project outcome (Momtaz 2020a, b).

#### Measures of success

There is still no consensus regarding a single success measure for ICO projects, since different studies follow different measures, each with a purpose and good reasoning to identify the success of a venture. In fact, some studies have aggregated several measures with similar results (de Jong et al. 2018). As the secondary market is seen as extremely important for the project to be successful, it is considered to be a measure of success because it is considered that the project’s success is directly linked to the tradability of tokens. Indeed, the project can only be considered successful if the tokens sold can be traded in a secondary market that takes place in a crypto exchange (Amsden and Schweizer 2019; Roosenboom et al. 2020). Other measures have also been developed and are equally important. One of these is a binary variable, in which a positive result is achieved when the project reaches its own soft-cap threshold (Roosenboom et al. 2020; Ahmad et al. 2020), and intrinsically related is the measure, in which a percentage is made on the capital reached above that threshold, with the most successful being those with higher percentages (de Jong et al. 2018). The same logic applies to a binary variable measuring the achievement of a pre-established hard cap (Ahmad et al. 2020). These measures can capture the success of the project in relative terms; in other words, in terms of its objectives. Hence, a project that predefines financing thresholds should be evaluated, taking them into account. However, these measures may not assess projects without financing thresholds.

Therefore, as in crowdfunding, the most common measure of success is the capital raised, allowing the inclusion of all the projects in a database and allowing their differentiation given the amount of capital they have achieved (Fisch 2019; Roosenboom et al. 2020; Šapkauskienė and Višinskaitė 2020). This measure lacks the advantage of being able to evaluate a project in relative terms. Nevertheless, the total capital raised is in line with the theory of open systems because it is the ultimate proof of information exchange and feedback. Furthermore, it allows the assessment of the final capital obtained, regardless of any predefined threshold, which should be a strong and unbiased measure for the quality of a given project. This should be the preferred measure for evaluating a project’s success.

#### Model and hypotheses

We built our research based on two main theories: general systems theory (Bertalanffy 1968) and signaling theory (Spence 1973). We also used the concept of adverse selection to complement the theoretical background (Grossman 1981). Our conclusion is that ICO projects fit the definition of an open systems model (Geiger et al. 2011a), in which there are several interactions between systems (supra-system relationships) and within themselves (sub-system relationships) (Mele et al. 2010). Therefore, there is a crucial importance given to the flow of information between the agents in the open systems model described (Bertalanffy 1968). This information is essential to the adaptation of the system via the feedback provided by an external system operating within the same environment, particularly the project team and investors. The ICO project environment is characterized by tremendous information asymmetries between the project’s promoters and investors (Momtaz 2019). The markets, in which the information is unequally distributed, tend to perform poorly (Leland and Pyle 1977), and another issue arises concerning the high likelihood of good-quality products being sold together with poor-quality products (Grossman 1981). This issue may also lead to dishonesty. The characteristics highlighted here are summarized in the concept of adverse selection.

For all of these reasons, the quality of the information provided as signals by the project’s promoters to investors is crucial (Milgrom 1981). We conclude that the information provided in the systems’ interaction contributes to the ultimate success of the project. This is verified by the effect of feedback provided by external systems and the subsequent adaptation of the project, as well as by the ultimate achievement of greater financing amounts (Skyttner 2005; Mele et al. 2010). We have highlighted that the information regarding these projects is considered to represent quality signals that help to reduce adverse selection and, ultimately, attract greater amounts of capital. We have summarized this information and considered it to comprise a project’s success factors. The literature highlights several success factors related to the project itself, the financing campaign, the use of social media, and the characteristics of human capital.

The main objective of the current research is to understand what the relevant success factors are, described here as signals, that contribute to the positive output of an ICO project. Therefore, the study tests the following hypotheses: (H1) the signals provided by the project promoters to investors in the open systems relationships contribute positively to the outcome of a project; (H2) projects that provide higher levels of quality information are more successful.

Four econometric models were built containing several variables identified as signals of project quality. The four models differ in terms of the dependent variable used: (i) logarithmic version of the total capital raised by a project, (ii) binary variable measuring the achievement of the soft-cap threshold, (iii) binary variable measuring the achievement of the hard-cap threshold, and (iv) binary variable measuring the existence of a secondary market. The reasoning behind the creation of several models was to ensure that the results remain consistent among them and to understand which dependent variable best captures a project’s success. The variables were clustered according to their characteristics and subsequently added to the model. We expect that projects that provide more information are also the most successful, measured in terms of capital raised. This measure is a direct result of good quality signaling and also functions as feedback in the dynamics of open systems.

## Methodology

### Database

The database was built through an API accessible with a premium subscription to the ICO Bench website and comprises 556 projects in the banking/financial sector. This sector was selected due to the impacts it faces from the rise of Fintech companies (Kou et al. 2021) and its role as a third party that is challenged by new models, such as ICOs (Frizzo-Barker et al. 2019; Campino et al. 2020). The database contains several key pieces of information on ICO projects: (i) information on the project itself, namely, the project’s year, information on the whitepaper, project ratings, and location; (ii) information on the campaign, namely, financing threshold amounts, the existence of bonus schemes or pre-sales of tokens; (iii) information on the team, namely, the number of team members, the number of advisors, and the number of projects, in which each member has participated; (iv) information on the use of social media, namely, which social media is used by the project and by the promoters.

Of the 556 projects available we were able to work with 428. The projects discarded had incomplete information, which prevented their correct analysis and could have led to a biased model. The sample is mainly comprised of projects located in Europe (239 projects). The second most represented location is the Asia–Pacific region with 106 projects, followed by North America with 44 projects. The other locations combined represent 59 projects. Most of the projects have low (180 projects) or average (179 projects) ratings attributed by external parties, and very few projects obtain exceptional classifications (69 projects). Most of the entrepreneurs have participated in up to three projects (3073 profiles), and only a few promoters have participated in more than three projects (85 profiles). The majority of the teams comprised between 11 and 20 members. Complementing the database, we collected information using the Twitter and LinkedIn social network platforms. We were able to collect information on Twitter activity, such as the number of followers and activity during the ICO campaign, and on LinkedIn networks, such as the team members’ number of connections from team members.

### Variables

The dependent variable that primarily captures projects’ success is the total capital raised by each project in U.S. dollars, as used in previous studies. We used its logarithmic form in our econometric model. We also regressed three extra models using different dependent variables: (i) a binary variable measuring the achievement of the soft-cap threshold, (ii) a binary variable measuring the achievement of the hard-cap threshold, and (iii) a binary variable measuring the existence of a secondary market. The independent variables can be divided into four main groups: (i) project variables (related to the project’s characteristics), (ii) campaign variables (related to ICO campaign characteristics), (iii) social network variables (related to the activity on social networks), and (iv) team variables (related to human capital characteristics).

Concerning the project variables, we captured variables related to the project itself and obtained the following: (i) whitepaper (we captured three main characteristics of the whitepaper, namely, its length, the disclosure of the project’s team, and technical aspects; (ii) restricted countries (number of countries, in which the project has restrictions); (iii) region (the project’s region divided between North America, Asia–Pacific, and Europe).

The variables capturing the campaign characteristics are focused on aspects relevant to the ICO campaign as follows: (i) pre-sales (captures the existence of token pre-sales); (ii) bonus scheme (captures the existence of a bonus to investors); (iii) token price (captures the price at which the token was sold); (iv) cryptocurrencies’ average prices (captures the yearly average price of Bitcoin and Ethereum); (v) currencies accepted (captures the number of currencies the project accepts as investment) (Table 1).

**Table 1:** Variable description

Variable	Description	**Source**
*Dependent variable*		
Log of the capital raised	Logarithm of the total capital raised in USD	ICO Bench
Soft-cap achievement	Binary variable measuring the achievement of the soft-cap threshold	ICO Bench
Hard-cap achievement	Binary variable measuring the achievement of the hard-cap threshold	ICO Bench
Existence of a secondary market	Binary variable measuring the existence of a secondary market	ICO Bench
*Project variables*		
Whitepaper: team disclosed	Binary variable stating the disclosure of the team in the whitepaper	Whitepaper
Whitepaper: technical	Binary variable stating the technicity of the whitepaper	Whitepaper
Whitepaper: word count log	Logarithm of the total whitepaper's word count	Whitepaper
Restricted countries	Variable accounting the number of countries where the project is restricted	ICO Bench
Region: North America	Binary variable stating the project's region	ICO Bench
Region: Asia–Pacific	Binary variable stating the project's region	ICO Bench
Region: Europe	Binary variable stating the project's region	ICO Bench
*Campaign variables*		
Pre-sales	Binary variable stating the existence of pre-sales	ICO Bench
Bonus scheme	Binary variable stating the existence of bonus schemes	ICO Bench
Token price log	Logarithm of the token's price	ICO Bench
Bitcoin price log	Logarithm of Bitcoin's average yearly price	CoinDesk
Ethereum price log	Logarithm of Ethereum's average yearly price	CoinDesk
Currencies accepted	Number of currencies accepted by the project	ICO Bench
*Social network variables*		
Twitter: active during campaign	Binary variable stating the campaign's status on Twitter	Twitter
Twitter: followers log	Logarithm of the number of Twitter's followers	Twitter
Twitter: number of tweets log	Logarithm of the number of tweets	Twitter
Github account	Binary variable stating the existence of a Github account	Github
Github: existing code prior to ICO	Binary variable stating the existence of code on Github prior to the ICO launching	Github
Website active on May, 2020	Binary variable stating the ICO website's status	ICO website
*Team variables*		
Team members log	Logarithm of the number of elements in the team	ICO Bench
Advisors log	Logarithm of the number of advisors in the team	ICO Bench
LinkedIn connections log	Team members' connections on LinkedIn	LinkedIn

Social networks have become an essential part of the promotion of new ventures, and we captured their characteristics as follows: (i) Twitter activity (activity during the campaign, the number of followers the project has, and the number of tweets made); (ii) GitHub activity (captures the existence of a GitHub account and the existence of publicly available code before the ICO campaign); (iii) active website (captures the existence of an available website in May 2020).

Concerning the team variables, we were able to capture the following aspects: (i) team members (number of members in the team); (ii) advisors (number of advisors in the project); (iii) LinkedIn connections (the sum of team members’ LinkedIn connections).

There were several more variables that we were able to capture, but that we decided not to include in the model due to multicollinearity issues (Wooldridge 2013). We obtained several ratings attributed to the project, namely, project rating, team rating, vision rating, and product rating. These variables had a strong relationship among themselves and, although for prediction purposes, this would not be an issue, collinearity could influence regression coefficients. Therefore, we decided to retain only the project rating because it is the most general rating that captures the greatest number of project features. The same occurred with Twitter followers and profiles followed by the project. A collinearity issue was present in this case, and we decided to keep only Twitter followers because, according to the literature, it is an important characteristic and, also, because it was considered to be a statistically significant variable with a higher coefficient.

### Methods

#### Data analysis

We used STATA 14 software to develop the econometric models and perform several tests. We first regressed the econometric model using the standard ordinary least squares (OLS) method and performed a test to detect skewness and kurtosis, which we verified to be present. Therefore, we applied a Shapiro–Wilk test to confirm that the residuals did not have a normal distribution (STATA 2020a). There was also an issue with heteroskedasticity, since the residuals exhibited non-constant variation confirmed by the Breusch-Pagan test and reinforced by White’s general test for heteroskedasticity, which overcomes some limitations of the first test (Williams 2020). We confirmed that there was no multicollinearity issue after adjusting for variables with a variance inflation factor (VIF).

#### Robust regression

The standard OLS method was used to regress the first and main econometric model, as its dependent variable is the logarithmic version of the total capital obtained by the project. Although the standard OLS method could be used, it could also be biased, and we decided to run a robust regression using the command “*rreg*” in STATA (STATA 2020b). Although the OLS estimator has dominated the literature and the application of regression techniques, robust regression techniques appeared as a strong substitute because they offer protection against distortion of anomalous data (Li 1985). There is no single method for a robust regression; on STATA, the command first fits the regression with Cook’s Distance excluding observations for which D > 1 and then works iteratively (Hilbe et al. 1991) by running a regression, calculating case weights from absolute residuals, and regressing again using those weights until the value of the weights drops below the predefined tolerance value of 0.01 (STATA 2020b). The weights are derived from two complementary weight functions, namely, Huber (1964) and biweights (Beaton and Tukey 1974). They are complementary measures since “Huber weights have problems dealing with severe outliers, whereas biweights sometimes fail to converge or have multiple solutions” (STATA 2020b).

This regression type has already been used in the ICO literature (de Jong et al. 2018; Fisch 2019). The data on ICO projects can be difficult to obtain because they are dispersed among several sources. Several websites have been collecting reliable data on these projects, which allows for their study. Nevertheless, ICOs are disruptive projects and a recent phenomenon that does not follow many specified rules. Therefore, the data on them are characterized by several outliers in several categories of analysis, which makes it harder to apply the standard OLS method for regression models. These reasons make the use of a robust regression for the analysis of these projects a strong solution to overcome normality issues, among others. In this study, both the standard OLS method and the robust regression method obtained similar results. We decided to present the robust regression results because they are more suitable for the type of data obtained for the reasons mentioned. Furthermore, we progressively added the variables to verify the coefficient and *p*-values behavior to check the robustness of the model.

#### Logistic regression

For the models based on binary dependent variables, a logistic regression was used, as it is the appropriate method for this type of variable (STATA 2020c). Standard r-squared and adjusted r-squared values are presented for the first model, which uses a robust regression method. However, for the remaining models using a logistic regression, a pseudo r-squared is presented, also known as McFadden’s pseudo-r-squared.

## Results

### Descriptive statistics

The projects comprising the database have a similar distribution in terms of success, considering the dependent variable measuring the total capital raised. However, using subsequent variables leads to a greater number of unsuccessful projects. Table 2 was built using IBM SPSS Statistics 26 to facilitate the analysis of the distribution of the independent variables contingent on the dependent variable used.

**Table 2:** Cross table between dependent and independent variables

	Capital raised in USD				Soft-cap achieved			
	Below/equal median	%	Above median	%	No	%	Yes	%
Project variables								
*Whitepaper: team disclosed*								
No	141	33	83	19	158	37	66	15
Yes	84	20	120	28	101	24	103	24
*Whitepaper: technical*								
No	208	49	136	32	229	54	115	27
Yes	17	4	67	16	30	7	54	13
*Whitepaper: word count*								
Below	136	32	78	18	151	35	63	15
Above	89	21	125	29	108	25	106	25
*Restricted countries median*								
Below	112	26	108	25	126	29	94	22
Above	113	26	95	22	133	31	75	18
*Region: North America*								
No	196	46	188	44	228	53	156	36
Yes	29	7	15	4	31	7	13	3
*Region: Asia–Pacific*								
No	172	40	150	35	195	46	127	30
Yes	53	12	53	12	64	15	42	10
*Region: Europe*								
No	115	27	94	22	130	30	79	18
Yes	110	26	109	25	129	30	90	21
Campaign variables								
*Pre-sales*								
No	100	23	96	22	113	26	83	19
Yes	125	29	107	25	146	34	86	20
*Bonus scheme*								
No	139	32	97	23	150	35	86	20
Yes	86	20	106	25	109	25	83	19
*Token price median*								
Below	112	26	102	24	132	31	82	19
Above	113	26	101	24	127	30	87	20
*BTC price median*								
< USD 1000	0	0	1	0	0	0	1	0
USD 1000–USD 5000	26	6	52	12	30	7	48	11
> USD 5000	199	46	150	35	229	54	120	28
ETH price median								
< USD 100	0	0	1	0	0	0	1	0
USD 100–USD 200	58	14	19	4	63	15	14	3
> USD 200	167	39	183	43	196	46	154	36
*CCY accepted median*								
Below	152	36	132	31	173	40	111	26
Above	73	17	71	17	86	20	58	14
Social network variables								
*Twitter active campaign*								
No	113	26	64	15	131	31	46	11
Yes	112	26	139	32	128	30	123	29
*Twitter followers median*								
Below	146	34	68	16	164	38	50	12
Above	79	18	135	32	95	22	119	28
*Twitter number of tweets*								
Below	143	33	72	17	159	37	56	13
Above	82	19	131	31	100	23	113	26
*Github account*								
No	125	29	83	19	138	32	70	16
Yes	100	23	120	28	121	28	99	23
*Github code prior ICO*								
No	159	37	113	26	176	41	96	22
Yes	66	15	90	21	83	19	73	17
*Website active on May, 2020*								
No	117	27	73	17	134	31	56	13
Yes	108	25	130	30	125	29	113	26
Team variables								
*Team members median*								
Below	138	32	77	18	149	35	66	15
Above	87	20	126	29	110	26	103	24
*Advisors median*								
Below	142	33	103	24	155	36	90	21
Above	83	19	100	23	104	24	79	18
*LinkedIn connections median*								
Below	134	31	80	19	148	35	66	15
Above	91	21	123	29	111	26	103	24
Total	225	53	203	47	259	61	169	39

	Hard-cap achieved				Existence of a secondary market			
	No	%	Yes	%	No	%	Yes	%
Project variables								
*Whitepaper: team disclosed*								
No	192	45	32	7	185	43	39	9
Yes	175	41	29	7	167	39	37	9
*Whitepaper: technical*								
No	306	71	38	9	298	70	46	11
Yes	61	14	23	5	54	13	30	7
*Whitepaper: word count*								
Below	190	44	24	6	183	43	31	7
Above	177	41	37	9	169	39	45	11
*Restricted countries median*								
Below	183	43	37	9	168	39	52	12
Above	184	43	24	6	184	43	24	6
*Region: North America*								
No	329	77	55	13	318	74	66	15
Yes	38	9	6	1	34	8	10	2
*Region: Asia–Pacific*								
No	278	65	44	10	265	62	57	13
Yes	89	21	17	4	87	20	19	4
*Region: Europe*								
No	179	42	30	7	174	41	35	8
Yes	188	44	31	7	178	42	41	10
Campaign variables								
*Pre-sales*								
No	153	36	43	10	146	34	50	12
Yes	214	50	18	4	206	48	26	6
*Bonus scheme*								
No	194	45	42	10	186	43	50	12
Yes	173	40	19	4	166	39	26	6
*Token price median*								
Below	190	44	24	6	180	42	34	8
Above	177	41	37	9	172	40	42	10
*BTC price median*								
< USD 1000	0	0	1	0	1	0	0	0
USD 1000–USD 5000	54	13	24	6	39	9	39	9
> USD 5000	313	73	36	8	312	73	37	9
*ETH price median*								
< USD 100	0	0	1	0	1	0	0	0
USD 100–USD 200	72	17	5	1	71	17	6	1
> USD 200	295	69	55	13	280	65	70	16
*CCY accepted median*								
Below	239	56	45	11	228	53	56	13
Above	128	30	16	4	124	29	20	5
Social network variables								
*Twitter active campaign*								
No	162	38	15	4	153	36	24	6
Yes	205	48	46	11	199	46	52	12
*Twitter followers median*								
Below	195	46	19	4	194	45	20	5
Above	172	40	42	10	158	37	56	13
*Twitter number of tweets*								
Below	196	46	19	4	192	45	23	5
Above	171	40	42	10	160	37	53	12
*Github account*								
No	182	43	26	6	172	40	36	8
Yes	185	43	35	8	180	42	40	9
*Github code prior ICO*								
No	238	56	34	8	229	54	43	10
Yes	129	30	27	6	123	29	33	8
*Website active on May, 2020*								
No	167	39	23	5	170	40	20	5
Yes	200	47	38	9	182	43	56	13
Team variables								
*Team members median*								
Below	186	43	29	7	177	41	38	9
Above	181	42	32	7	175	41	38	9
*Advisors median*								
Below	204	48	41	10	197	46	48	11
Above	163	38	20	5	155	36	28	7
*LinkedIn connections median*								
Below	180	42	34	8	178	42	36	8
Above	187	44	27	6	174	41	40	9
Total	367	86	61	14	352	82	76	18

Note that the results are very similar between the first two models (capital raised and soft-cap achievement) and between the last two (hard-cap achievement and secondary market existence), but with substantial differences among them. In the last two models, the percentage of unsuccessful projects is high, which makes the analysis much more difficult. The measures of success used in the first two models allowed us to obtain a much more balanced percentage between success and failure, maintaining a clear tendency for more unsuccessful projects. The last two variables seem to incompletely capture the success of a project for several reasons: (i) the achievement of the hard cap assumes that the projects have a maximum financing threshold, which is not always the case (the same issue exists for the soft-cap achievement variable); (ii) the achievement of the hard-cap threshold is not a necessary condition for the project to be successful because a project has the necessary conditions to work once it achieves the soft-cap threshold; (iii) although it is expected, several projects may not achieve the stage of tradability in a secondary market, but that does not necessarily mean that they did not achieve the financing thresholds needed for the project’s funding.

Focusing on the first two models, which allow for clearer conclusions regarding descriptive statistics, we confirm that disclosing the team and having a technical and longer whitepaper are associated with success, accounting for the highest percentage of success. In terms of country restrictions and project region, there is no clear division between successful and unsuccessful projects, as the percentages are similarly divided and do not lead to a clear conclusion. Concerning the campaign variables, we cannot reach a clear conclusion with descriptive statistics on the variables related to the existence of pre-sales, the number of currencies accepted by the project, or the token price. This is due to a similar allocation of successful and unsuccessful projects, regardless of the value of this variable.

The prices of cryptocurrencies, namely, Bitcoin and Ethereum, seem to be inversely related to the project’s success. Whereas a cheaper price for Bitcoin is related to more successful projects, the opposite occurs with Ethereum, which tends to have more successful projects when its price is higher. The variables related to the use of social networks tended to influence project success. Having an active Twitter account during the ICO campaign is associated with successful projects (32%), in contrast to not having an active Twitter campaign (15%), and having more followers is linked to successful projects (32%), in contrast to having smaller networks (16%). The number of tweets does not allow for a clear analysis because of the percentages obtained. Although at a much smaller scale, we also confirm that having an active GitHub account is linked to greater success (28%) than not having one (19%). Having code publicly available before the start of the ICO campaign is not confirmed as having a strong relationship with success, contrary to the existence of an active website, because more projects are considered successful when they have one (30%) than when they do not (17%). Finally, the team variables considered relevant to a project’s success are the number of team elements and LinkedIn networks. Larger teams and networks have higher percentages of successful projects (29% for both) than the opposite (18% and 19%, respectively).

As shown in Table 3, significant discrepancies were found in the whitepaper word count. There were whitepapers with zero words because they were not found or did not exist, and there were whitepapers with as many as 88,211 words. The mean value was 6,465 words. The same happens with data concerning restricted countries, which can range from zero to 124, with a small mean for the two countries.

**Table 3:** Descriptive statistics

	Descriptive statistics				
	Observations	S.D	Min	Max	Mean
Whitepaper word count	428	6738.09	0	88,211	6464.51
Restricted countries	428	6.47	0	124	1.56
Token price log	428	0.42	0	3.48	0.21
Bitcoin price log	428	0.12	2.75	3.88	3.82
Ethereum price log	428	0.20	1.03	2.68	2.54
CCYs accepted	428	2.02	1	30	2.23
Twitter: followers log	428	1.52	0	5.45	2.46
Twitter: number of tweets log	428	1.14	0	3.85	1.72
Team members	428	8.22	1	47	12.89
Advisors log	428	0.35	0	1.23	0.34
LinkedIn connections log	428	1.47	0	4.24	2.71

To normalize the data, we rescaled several variables using log transformation. We also developed a frequency table for binary variables, as shown in Table 4. We can verify that, although balanced, most whitepapers do not disclose the team (52%), and the large majority are not technical (80%). The predominant region for a project’s location was Europe (51%).

**Table 4:** Frequencies

	Frequencies table		
	Frequency	Percentage	Cumulative percentage
*Whitepaper: team disclosed*			
No	224	52	52
Yes	204	48	100
Total	428	100	–
*Whitepaper: technical*			
No	344	80	80
Yes	84	20	100
Total	428	100	–
*Region: North America*			
No	384	90	90
Yes	44	10	100
Total	428	100	–
*Region: Asia–Pacific*			
No	322	75	75
Yes	106	25	100
Total	428	100	–
*Region: Europe*			
No	209	49	49
Yes	219	51	100
Total	428	100	–
*Pre-sales*			
No	196	46	46
Yes	232	54	100
Total	428	100	–
*Bonus scheme*			
No	236	55	55
Yes	192	45	100
Total	428	100	–
*Twitter active campaign*			
No	177	41	41
Yes	251	59	100
Total	428	100	–
*Github account*			
No	208	49	49
Yes	220	51	100
Total	428	100	–
*Github code prior ICO*			
No	272	64	64
Yes	156	36	100
Total	428	100	–
*Website active on May, 2020*			
No	190	44	44
Yes	238	56	100
Total	428	100	–

Although the majority of the projects included pre-sales of tokens (54%), most of them had no bonus scheme (55%). In terms of variables related to the use of social networks, we verified that most projects had an active campaign on Twitter (59%). The projects with a GitHub account represent 51% of the sample, and only 36% of them had publicly available code prior to the ICO campaign. Currently, 56% of projects have active websites.

After performing a correlation and VIF analysis,^1^ we confirmed that there were no collinearity issues. As previously discussed in the methodology section, we discarded four variables that showed high VIF values (higher than 10), which could compromise the analysis, particularly concerning the model regressed with the standard OLS method. These variables were clearly correlated among them, namely, the ratings attributed to different aspects of the project, Twitter profile followers, and following of the project. After reducing the number of variables, we obtained VIF values with a mean of 1.90.

### Econometric model

We started by regressing the econometric model with the dependent logarithmic variable (log of the total capital obtained) using the standard OLS method^2^ and, due to data limitations, regressed a second model using the robust regression in STATA. As expected, we obtained very similar results, regardless of the method used. Although the R-squared and adjusted R-squared measures are not the most appropriate for application to a robust regression, we present them and use them because they are consistent with the values obtained when the standard OLS method was used. We obtained a final R-squared of 0.32 for the OLS model and 0.33 for the robust regression, as well as a final adjusted R-squared of 0.28 for the OLS model and 0.29 for the robust regression. These measures increase with the inclusion of further independent variables that progressively contribute to the variance of the dependent variable. Along with the inclusion of new variables in both models, the already existing ones keep their significance and new ones are added, which can also be considered statistically significant. Furthermore, we developed three logistic regressions with dependent binary variables: (i) a binary variable measuring the achievement of the soft-cap threshold, (ii) a binary variable measuring the achievement of the hard-cap threshold, and (iii) a binary variable measuring the existence of a secondary market. The results are listed in Table 5.

**Table 5:** Econometric models

	Model 1				Model 2			
	Dependent variable: capital raised in USD log				Dependent variable: binary variable of soft-cap achievement			
	Project variables	+ Campaign variables	+ Social networks variables	+ Team variables	Project variables	+ Campaign variables	+ Social networks variables	+ Team variables
R^2^ (Pseudo R^2^)	0.16	0.26	0.30	0.33	0.09	0.16	0.21	0.22
Adjusted R^2^	0.15	0.24	0.27	0.29	–	–	–	–
Observations	428	428	428	428	428	428	428	428

	Coefficient	SE	Coefficient	SE	Coefficient	SE	Coefficient	SE	Coefficient	SE	Coefficient	SE	Coefficient	SE	Coefficient	SE
*Project variables*																
Whitepaper: team disclosed	1.31	0.39**	1.31	0.36***	1.27	0.36***	1.02	0.36**	0.64	0.23*	0.88	0.26**	0.90	0.27**	0.81	0.28**
Whitepaper: technical	3.00	0.44***	2.73	0.41***	2.54	0.41***	2.35	0.41***	1.10	0.27***	1.06	0.28***	1.01	0.30**	0.93	0.30**
Whitepaper: word count log	0.10	0.13	0.05	0.12	− 0.05	0.12	− 0.04	0.12	0.15	0.09*	0.13	0.09	0.07	0.10	0.05	0.10
Restricted countries	− 0.00	0.02	0.12	0.06*	0.00	0.02	− 0.00	0.02	− 0.01	0.02	0.00	0.01	− 0.00	0.01	− 0.00	0.02
Region: North America	− 0.44	0.70	− 0.89	0.64	− 0.85	0.64	− 0.82	0.63	− 0.46	0.44	− 0.99	0.49**	− 0.96	0.51*	− 0.87	0.52
Region: Asia–Pacific	0.53	0.57	0.63	0.52	0.51	0.51	0.37	0.50	0.07	0.35	0.02	0.37	− 0.03	0.38	− 0.04	0.39
Region: Europe	0.71	0.51	0.55	0.47	0.39	0.46	0.38	0.46	0.21	0.31	0.04	0.33	− 0.01	0.35	− 0.02	0.35
*Campaign variables*																
Pre-sales			− 0.16	0.32	− 0.15	0.32	− 0.26	0.31			− 0.23	0.23	− 0.24	0.24	− 0.32	0.24
Bonus scheme			0.92	0.33**	0.90	0.33**	0.88	0.33**			0.44	0.23*	0.45	0.24*	0.41	0.25
Token price log			0.70	0.38*	0.70	0.37*	0.00	0.00*			0.23	0.27	0.33	0.27	0.00	0.00
BTC price log			− 10.00	1.82***	− 9.81	1.76	− 10.18	1.72***			− 7.51	1.43***	− 7.50	1.48***	− 7.71	1.47***
ETH price log			3.82	1.03***	3.88	1.01***	3.80	1.00***			2.31	0.84**	2.65	0.88**	2.55	0.88**
CCY accepted			0.06	0.08	0.05	0.08***	0.04	0.07			− 0.00	0.05	− 0.01	0.05	− 0.02	0.05
*Social network variables*																
Twitter active campaign					0.71	0.43	0.79	0.43*					0.80	0.33**	0.79	0.33**
Twitter followers log					0.38	0.20*	0.38	0.20*					0.32	0.14**	0.35	0.15**
Twitter number of tweets log					− 0.23	0.29	− 0.29	0.29					− 0.28	0.21	− 0.34	0.22
Github account					0.34	0.45	0.15	0.45					0.30	0.34	0.21	0.35
Github code prior ICO					0.19	0.47	0.24	0.46					− 0.01	0.34	− 0.02	0.35
Website active on May, 2020					0.38	0.34	0.31	0.34					0.26	0.26	0.18	0.26
*Team variables*																
Team members							2.04	.69**							0.83	0.55
Advisors log							− 0.06	0.49							− 0.05	0.37
LinkedIn connections log							− 0.04	0.13							0.08	0.11

	Model 3				Model 4			
	Dependent variable: Binary Variable of Hard-cap Achievement				Dependent variable: binary variable for the existence of a secondary market			
	Project variables	+ Campaign variables	+ Social networks variables	+ Team variables	Project variables	+ Campaign variables	+ Social networks variables	+ Team variables
R^2^ (Pseudo R^2^)	0.06	0.17	0.21	0.24	0.07	0.19	0.22	0.22
Adjusted R^2^	–	–	–	–	–	–	–	–
Observations	428	428	428	428	428	428	428	428

	Coefficient	SE	Coefficient	SE	Coefficient	SE	Coefficient	SE	Coefficient	SE	Coefficient	SE	Coefficient	SE	Coefficient	SE
*Project variables*																
Whitepaper: team disclosed	− 0.38	0.30	0.01	0.33	− 0.06	0.35	− 0.07	0.37	− 0.14	0.29	0.32	0.33	0.19	0.35	0.21	0.35
Whitepaper: technical	0.94	0.31**	0.89	0.35**	0.77	0.38**	0.80	0.39**	1.36	0.30***	1.22	0.33***	1.05	0.35**	1.04	0.35**
Whitepaper: word count log	0.25	0.13*	0.30	0.14**	0.31	0.15**	0.33	0.16**	0.01	0.10	0.01	0.12	− 0.05	0.12	− 0.03	0.12
Restricted countries	− 0.05	0.06	− 0.01	0.03	− 0.02	0.04	− 0.01	0.03	− 0.01	0.04	0.01	0.01	0.01	0.02	0.01	0.02
Region: North America	0.23	0.61	− 0.25	0.68	− 0.31	0.70	− 0.36	0.75	1.12	0.58*	0.65	0.67	0.67	0.67	0.69	0.68
Region: Asia–Pacific	0.43	0.49	0.64	0.54	0.52	0.55	0.51	0.58	0.70	0.51	0.88	0.58	0.81	0.58	0.80	0.58
Region: Europe	0.37	0.46	0.50	0.50	0.36	0.50	0.58	0.54	0.92	0.48*	1.02	0.56*	1.00	0.55*	0.98	0.55*
*Campaign variables*																
Pre-sales			− 1.06	0.33**	− 1.06	0.34**	− 1.02	0.35**			− 0.72	0.30**	− 0.74	0.30**	− 0.75	0.31**
Bonus scheme			− 0.21	0.34	− 0.24	0.35	− 0.05	0.36			− 0.15	0.30	− 0.14	0.32	− 0.16	0.32
Token price log			0.67	0.29**	0.76	0.30**	0.01	0.00**			− 0.32	0.37	− 0.28	0.39	− 0.01	0.01
BTC price log			− 4.43	1.78*	− 4.46	1.86**	− 4.33	1.90**			− 7.66	1.74***	− 7.33	1.77***	− 7.35	1.76***
ETH price log			0.85	1.23	0.96	1.26	0.81	1.29			1.41	1.22	1.77	1.23	1.75	1.23
CCY accepted			− 0.08	0.10	− 0.12	0.10	− 0.14	0.11			− 0.03	0.07	− 0.05	0.08	− 0.05	0.08
*Social network variables*																
Twitter active campaign					0.45	0.46	0.42	0.47					− 0.37	0.42	− 0.36	0.42
Twitter followers log					0.41	0.21*	0.42	0.22*					0.21	0.19	0.21	0.19
Twitter number of tweets log					− 0.14	0.28	− 0.05	0.31					0.10	0.27	0.08	0.28
Github account					0.15	0.49	0.19	0.53					− 0.49	0.50	− 0.43	0.51
Github code prior ICO					0.14	0.49	0.26	0.52					0.71	0.51	0.63	0.51
Website active on May, 2020					− 0.56	0.36	− 0.61	0.38					0.49	0.34	0.47	0.35
*Team variables*																
Team members							0.98	0.75							− 0.12	0.69
Advisors log							− 0.28	0.53							− 0.22	0.47
LinkedIn connections log							− 0.40	0.13*							0.09	0.13

Significance levels: *p* < 0.01 (***); *p* < 0.05 (**); *p* < 0.1 (*)

In the first two models, the results are consistent in terms of the statistical significance of the variables and coefficients. We obtain statistical significance for the following project variables: (i) whitepaper (team disclosed) and (ii) whitepaper (technical). The campaign variables considered significant are as follows: (i) bonus scheme, (ii) token price, (iii) Bitcoin price, and (iv) Ethereum price. Concerning the social network variables, the ones considered significant are: (i) Twitter active during the ICO campaign and (ii) Twitter number of followers. The team variable considered significant was the number of team members. Although statistically insignificant, we would like to highlight the importance of Twitter activity measured by the number of Tweets, since, as in the literature, we found that extremely active Twitter accounts, which put pressure on investors, may contribute negatively to project success.

Even though there are some discrepancies between the results of Models 3 and 4, there are variables that retain their strong statistical significance in all the models: (i) whitepaper technical, (ii) bitcoin price, and (iii) number of Twitter followers (except for Model 4). Model 2 provides an interesting conclusion regarding the length of the whitepaper, revealing that it contributes to the project’s success, mainly due to the amount of information included, which is important for reducing information asymmetries. Model 4 contributes to the discussion by revealing the importance of locations concerning projects located in Europe. Models 3 and 4 prove to be statistically significant for the variable measuring the existence of pre-sales and reveal its negative impact on the success of a project. It is important to highlight that the coefficients are consistent among the models, but it was not possible to maintain similar results regarding the statistical significance.

## Conclusions and discussion

### Results discussion

We built on systems theory and signaling theory as a theoretical background for the current research. The ICO projects were interpreted in light of the theoretical framework proposed by both theories. It was found that the ICO projects operate as a system, particularly as an open system (Geiger et al. 2011a), which develops relationships with others within the same environment (Doan et al. 2011). The exchange of information is crucial in this particular framework, and the subsequent feedback allows the system to adapt correctly (Bertalanffy 1968). However, the exchange of information is of greater importance in markets with high information asymmetries between entrepreneurs and investors (Spence 1973). As the ICO market is characterized by such asymmetries (Momtaz 2019), the creation of quality signals by the project’s promoters that certify the quality of their venture becomes even more important. These signals and feedback are exchanged in this supra-system relationship (Geiger et al. 2011a) and can, ultimately, lead to a successful project capable of obtaining higher financing amounts (Fisch 2019).

To assess the factors influencing the success of a project, we defined the most appropriate measure as the total capital raised by a given venture (Fisch 2019; Šapkauskienė and Višinskaitė 2020). Thus, we first regressed a model using the standard OLS method and, thereafter, a model using a robust regression method, owing to data limitations. The results were identical regardless of the method used. We further expanded our analysis by building three additional models using a logistic regression, for which the dependent variables were: (i) a binary variable measuring the achievement of the soft-cap threshold (de Jong et al. 2018); (ii) a binary variable measuring the achievement of the hard-cap threshold; (iii) a binary variable measuring the existence of a secondary market (Amsden and Schweizer, 2019). These measures may also be considered as accurate measures of success, especially the binary variable measuring the achievement of the soft-cap threshold. The soft-cap threshold is the minimum amount that the project is willing to take, in order to proceed. Therefore, it is considered that a project can start after this goal is achieved. Regarding the achievement of the hard-cap threshold, the logic behind it is similar to the achievement of the soft-cap threshold. Nevertheless, this is the maximum amount a project is willing to take, and it does not necessarily need to achieve it to be considered successful. The tradability of the token in a secondary market is also considered desirable and a sign of a project’s success. Nonetheless, a project might not need to have its token traded in a secondary market for it to be considered successful. Consequently, we find the most suitable measure for the success of a project to be the total capital raised, similar to the achievement of the minimum soft-cap threshold established.

The results were similar in the models using the total capital raised and soft-cap achievement as the dependent variables. The same happens for the models using the hard-cap achievement and the existence of a secondary market. Although the coefficients remain stable regardless of the dependent variable used, the statistical significance changes.

Regardless of the model used, we confirmed that the whitepaper has become a crucial part of ICO projects, in order to reduce information asymmetry. We conclude that the length of the whitepaper can positively help inform investors about the project, as confirmed in Model 3. Investors tend to pay closer attention to the content of the whitepaper (Adhami et al. 2018), such as the disclosure of team members and technical details (Feng et al. 2019; Fisch 2019). These two variables have positive coefficients, and the variable measuring the technicality of the whitepaper is approved in all models. The variable measuring the disclosure of the team was approved in the first two models.

Our conclusions do not confirm the argument that bonus schemes negatively affect project success. In our analysis, we have a positive influence of bonus schemes for ICOs in the banking/financial sector in the first model. Furthermore, our analysis reveals that higher token prices are linked to more successful projects. Our research suggests that investors prefer more expensive tokens with bonus schemes that allow them to overcome higher prices, which are perceived as a signal of good quality. As per our analysis, cheaper tokens are less successful and can be considered as scams, while the opposite occurs with higher token prices.

We confirmed the dependency of ICO projects on the cryptocurrency market. The prices of cryptocurrencies are also linked to a project’s success, as confirmed in our research and in the literature (Myalo and Glukhov 2019). We found a negative impact of higher prices of Bitcoin on project success (confirmed in all models). On the contrary, higher Ethereum prices meant more successful projects (not confirmed in the last two models). As most ventures are Ethereum-based (Fenu et al. 2018), the appreciation of cryptocurrency might influence investors perception of an ICO as a good investment.

Our research also shows a relationship between the use of Twitter and a project’s outcome. Having an active ICO campaign on Twitter has a strong impact on the project’s success, as well as the network of followers being a way of reducing information asymmetries and keeping potential investors informed (Burns and Moro 2018; Cerchiello et al. 2019; Fisch 2019; Xuan et al. 2020). The literature indicates that larger teams tend to be more successful (Ante 2018; Amsden and Schweizer 2019; Cerchiello et al. 2019; Giudici and Adhami 2019; Liu and Wang 2019; Roosenboom et al. 2020). We were also able to confirm this with our analysis in Model 1, revealing that larger teams have more chances to be heterogeneous and have more diverse inputs, creating valuable human capital.

In conclusion, this research was able to confirm both the proposed hypotheses. The first hypothesis, which states that the quality signals provided by the project promoters to the investors in the open system relationships contribute positively to the outcome of a project, is confirmed by the statistical significance and coefficients of several variables in the econometric model. For instance, we confirm that the signal of expensive tokens complemented by a bonus scheme attracts investors, as does the existence of a larger and heterogeneous team capable of producing positive results when compared to small and homogeneous teams. The second hypothesis, which states that projects that provide higher levels of quality information are more successful, is also supported by the analysis of whitepaper and social network variables. Consequently, the projects that disclose the most information in the whitepaper and have comprehensive documents are more likely to succeed. Similarly, projects that continuously inform stakeholders through social networks (being active during the campaign) and keep them engaged (measured by the number of followers) are also more capable of achieving positive results.

### Theoretical contributions

The findings of our study contribute to the literature on ICO projects. We were able to assemble a database and test most of the variables concerning the success factors of ICOs mentioned in the literature. We confirmed several aspects present in the literature, but also obtained different results, thereby contributing to a wider discussion. Our research reported here, based on a considerably large database of blockchain projects and the use of theory, makes our contribution unique in the literature.

Furthermore, this research has elaborated on systems theory and signaling theory. These theoretical backgrounds were adapted to the blockchain-based projects, and we were able to confirm that ICOs are open systems operating in an environment with several other systems that interact among them. The importance of feedback and the exchange of information is crucial in systems with these characteristics. Nevertheless, the ICO markets are characterized by information asymmetries between project promoters and investors, which can lead to flawed markets. This is why providing quality signals, here described as success factors, created by the project and sent to investors, are so important.

We were able to prove that the two theories we used are relevant in the context of ICOs and that further analysis on them should account for the theoretical foundations analyzed here. Thus, the findings of this study uniquely contribute to the literature by using and interconnecting the two theories and adapting them to innovative blockchain projects. This provides ICO projects with an appropriate theoretical framework for their analysis.

### Managerial implications

Our research is also important for regulators, mainly regarding our conclusions concerning whitepapers. Initial coin offering projects are highly unregulated, but because of the amount of money involved and the importance they have acquired, regulators need to pay closer attention to them. As the current risks of blockchain projects involve security and fraud, regulators should focus on measures supporting transparency and accountability to avoid fraud and crime (Frizzo-Barker et al. 2019). The whitepaper has been a way of reducing the lack of information in ICO projects and works as a self-regulated prospectus with crucial information on the project. As per our research, there is strong evidence that a professional whitepaper that discloses important information is essential to the project’s success. These conclusions might be important in guiding future regulator actions.

For the promoters of ICO projects, the implications of this research are mainly related to investors’ perceptions of the project and the aspects promoters should pay particular attention to when promoting their ideas. We reinforce some of the conclusions found in the literature and add new insights that can be important in determining a project’s outcome. Indeed, a project promoter should, for instance, focus on having a detailed whitepaper, in which the team is disclosed and some technical details are highlighted. Furthermore, the project’s timing should be selected to take advantage of cryptocurrency prices, the campaign should be as short as possible, and social networks should be well-managed. In summary, the ICO project’s promoters should take advantage of the messages presented in the literature to control all the factors that can obtain the best outcome.

### Main limitations

The database used for this research originally comprised 558 ICO projects, but some had to be discarded due to lack of information, leaving 428 projects for analysis. The database included only projects in the banking/financial sectors and may, therefore, not be generalizable to other industries. We also removed some of the variables collected because of their collinearity levels. It would be valuable to keep these variables in future studies, in order to clearly observe their behavior. Concerning Twitter and GitHub activity, we faced an issue with inactive or blocked accounts. Although the number was not enough to compromise the research, we point out the fact that, in some cases, we were not able to collect information on social platform activity, due to the current unavailability of the account. The same issue occurred with some projects’ whitepapers, which we could not find, even by searching several platforms dedicated to ICOs and on the project’s official website.

We collected information on the ratings attributed to the project, namely, project rating, vision rating, and team rating. We were not able to use all of these factors because of their degree of collinearity. We decided to remove all these variables because we could not be certain of the time when they were attributed. The ratings are usually attributed before the ICO starts and are updated throughout the campaign and again after it is concluded. Although we are confident of their correct usage in the model, we could not ensure that they were completely unbiased and we, therefore, decided to exclude them. In our preliminary analysis, we concluded that the ratings for the team, project, and product had a positive impact on project success, and the rating for the vision had a negative impact on the project. This is due to the fact that visionary projects tend to be more difficult to achieve and harder to understand, creating a barrier for investment (Gompers and Lerner 2001). Other studies have confirmed this conclusion (Fenu et al. 2018; Rhue 2018; Lee et al. 2019; Xuan et al. 2020).

A final limitation identified in this research concerns the inclusion of variables measuring the achievement of soft-cap and hard-cap thresholds. To keep the same sample used for the remaining models, we considered the value 0 for the projects that did not establish fundraising thresholds. Their exclusion would reduce the sample, as several projects do not define financing thresholds, and after regressing the model with a reduced sample, we concluded that the results remain consistent with those obtained when using the entire sample collected.

### Avenues for future research

There are many avenues for future research in ICO projects because of the novelty of the topic and several unexplored features. After performing the current research, we found that some variables have a strong impact on project success, for instance, the whitepaper. For future studies, it would be interesting to isolate these variables and focus on their effects. Regarding the whitepaper, it would be interesting to analyze its smallest aspects to understand what is most important. Furthermore, we confirmed that the secondary market is of great importance. Therefore, it is important to analyze the behavior of tokens in the secondary market in terms of volatility, tradability, returns for investors, and many other factors. Concerning the database, it would be useful to construct a wider database and cluster it by the project’s industry before determining the most successful industry and confirming the success factors that are transversal to industries or those that apply to only one specific industry.

Building on OST and signaling theory, future studies might focus on the sub-system relationships, that is, those between projects, to determine if there is any degree of cooperation and the extent to which cooperation is useful. It would also be interesting to analyze the relationships within the project, such as human relationships, aspects of corporate governance, or the way feedback is processed for the correct adaptation of the system (Mele et al. 2010).

## Data Availability

The datasets used and/or analyzed in the current study are available from the corresponding author on reasonable request.

## Footnotes

1 Detailed results are available from the authors upon request.

2 Detailed results available from the authors upon request.
